# The Clinical Assessment of MicroRNA Diagnostic, Prognostic, and Theranostic Value in Colorectal Cancer

**DOI:** 10.3390/cancers13122916

**Published:** 2021-06-11

**Authors:** Hussein Al-Akhrass, Niki Christou

**Affiliations:** 1 Turku Bioscience, Tykistökatu 6, FI-20520 Turku, Finland; Hussein.al-akhrass@utu.fi; 2 Digestive Surgery Department, University Hospital Limoges, Avenue Martin Luther King, CEDEX 1, 87042 Limoges, France; 3 EA3842 CAPTuR Laboratory “Cell Activation Control, Tumor Progression and Therapeutic Resistance”, Faculty of Medicine, 2 rue du Docteur Marcland, CEDEX 1, 87025 Limoges, France; 4 Colorectal Surgery Department, University Hospitals Birmingham NHS Foundation Trust, Birmingham B152TH, UK

**Keywords:** MiRNA, colorectal cancer, biomarker, diagnostic, prognosis

## Abstract

**Simple Summary:**

MiRNAs are of great interest within colorectal cancers in diagnosis, prognosis, and within the field of personalized treatments; they are present within different biological fluids such as blood and can lead to specific information for daily clinical use. Herein, we review the current literature focusing on miRNAs as potential diagnostic and prognostic biomarkers in patients treated for colorectal cancers. Detection and analysis of miRNA expression are cost-effective and lead to high sensitivity and specificity rates. However, it is now necessary to highlight the most sensitive and specific miRNAs for each goal, either diagnostic, prognostic, or theranostic, thanks to multicentric prospective studies.

**Abstract:**

MiRNAs have recently become a subject of great interest within cancers and especially colorectal cancers in diagnosis, prognosis, and therapy decisions; herein we review the current literature focusing on miRNAs in colorectal cancers, and we discuss future challenges to use this tool on a daily clinical basis. In liquid biopsies, miRNAs seem easily accessible and can give important information toward each step of the management of colorectal cancers. However, it is now necessary to highlight the most sensitive and specific miRNAs for each goal thanks to multicentric prospective studies. Conclusions: by their diversity and the feasibility of their use, miRNAs are getting part of the armamentarium of healthcare management of colorectal cancers.

## 1. Introduction

Colon and rectal cancers (CRCs) are respectively the 5th and 8th cancers in terms of incidence worldwide, and they represent the fourth most cancer-related deaths [1]. The most frequent histological type is Liberkhünien adenocarcinomas. The carcinogenesis of such tumors can be explained by several mutational and signaling network alterations. For instance, we can quote different alterations of oncogenes or tumor suppressor genes linked to signaling pathway changes such as within the Wnt (portmanteau created from the names Wingless and Int-1) [2] signaling pathway but also receptor tyrosine kinase pathways leading to cellular proliferation, DNA repair, cell cycle arrest, and apoptosis pathway inactivation [3]. 

Currently, miRNAs have been an ever-increasing topic of interest. MiRNAs belong to the RNA interference family originally discovered in 1998 by Andrew Fire and Craig Mello [4] (2006 Nobel Prize laureates in Physiology or Medicine). Their analysis is cost-effective and encompasses high levels of both sensitivity and specificity. They are short non-coding strands of RNA comprising around 19 to 22 nucleotides [5]. Unsurprisingly, miRNAs play a fundamental role in the regulation of physiological processes such as embryogenesis [6] as well as several human pathologies such as cancer, auto-immune, and cardiovascular diseases [7,8,9,10]. Until now, around 2600 mature miRNAs in the human genome have been discovered and are accessible through publicly available databases such as miRBase v.22 [11]. MiRNAs exhibit a broad regulatory potential since more than 60% of protein-coding mRNAs contain highly conserved miRNA-binding sequences [12]. Generally, miRNAs inhibit gene expression through binding to the untranslated region within the 3’ end of messenger RNAs (mRNAs) [13].

Different publications have shown miRNA release into fluids such as stools [14] and blood [15]. Extracellular miRNAs can be transported to target cells through several transport pathways. We can quote extracellular vesicles, such as exosomes, or the mechanism by interaction with proteins like argonaute 2 (Ago2) as described in human plasma analysis [15], high density lipoprotein (HDL), and nucleophosmin 1 (NPM 1). These associations are essential for them in order to avoid any digestion from RNase [16]. It is worth noting that the carrier of one miRNA may not be the only one. Furthermore, different carriers can exist for one miRNA and depend on the tissue specificity and cell type that are going to be targeted by the miRNA [17].

Here, we briefly introduce the biosynthesis of miRNAs, and we review the current clinical achievements related to their potential roles as diagnostic and prognostic biomarkers in CRCs.

## 2. Canonical miRNA Biosynthesis

The canonical miRNA biosynthesis pathway begins with the transcription of a primary miRNA (pri-miRNA) forming a hairpin structure. Pri-miRNA is subsequently processed into an miRNA precursor (pre-miRNA) by the nuclear microprocessor complex formed by the RNA-binding protein DiGeorge Critical Region 8 (DGCR8) and the ribonuclease 3 enzyme Drosha [18]. This step results in the cleavage of pri-miRNA at the base of the hairpin structure. Exportin-5 transports the pre-miRNA to the cytoplasm where it is processed by the RNase III endonuclease Dicer that eliminates the loop sequence, thus resulting in a mature miRNA duplex [19,20]. The directionality of each strand determines its name. Mature miRNAs 5p and 3p arise from the 5′ and 3′ ends, respectively. Ultimately, the miRNA duplex is unwound, and the remaining single-stranded miRNA is loaded into the Argonaute complex in an ATP-dependent manner, forming a central component of the RNA-induced silencing complex (RISC) [21,22]. It is worth noting that multiple Drosha/DGCR8- and Dicer-independent biosynthesis pathways can generate non-canonical miRNAs (reviewed in [23]).

## 3. RNAs Regulate Fundamental Processes of CRC Growth

In this section, we highlight the history of miRNA research in relation to CRCs and we review key fundamental roles of miRNAs in regulating CRC signaling. Without being exhaustive, we provide a focus on signaling pathways frequently deregulated in CRCs and underpinning essential processes for CRC progression such as the epithelial to mesenchymal transition (EMT), cancer angiogenesis, and uncontrolled cell division.

First of all, it is important to mention that colon and rectal cancer arise from different genetic and epigenetic alterations within normal colonic and rectal tissues. At the molecular scale, the development of an adenoma followed by apparition of a CRC corresponds to the progressive accumulation of gene mutations within the nucleus of colonic epithelial cells: activation of oncogenes and inactivation of tumor suppressor genes.

There are two main pathways of colorectal carcinogenesis that result from genetic instability: one, the most common, is detected at the chromosomal level (chromosomal instability, “CIN”); the second is at the nucleotide level (instability of the loci of microsatellites). These two distinct pathways result in morphologically similar lesions (adenomas) but with a different progression to cancer, more important in the path of micro satellite instability. 

Regarding “CIN” tumors, they present a characteristic set of mutations on the tumor suppressor gene *APC* as well as on the proto-oncogene Kirsten RAS (*KRAS*), activating the initiation and progression signaling pathways of CRC [24]. In 2008, Nagel et al. showed that miR-135a&b was able to target the 3′ untranslated region of *APC*. This led to the abolishment of its expression, with induction of downstream *Wnt* pathway activity [25]. Wang et al., in 2016, proved that miR-384 repressed *KRAS* expression by directly targeting its 3’untranslated region [26]. 

The second pathway of colorectal carcinogenesis is the molecular mechanism concerning 15% of sporadic CRCs and is typically observed in Lynch syndrome (HNPCC: 3% of cases). These cancers are called replication errors (RER+) or microsatellite instabilities (MSI). Cancer cells have normal DNA content (diploidy) and have no chromosomal loss but have abnormalities in DNA mismatch repair (MMR) genes. The components of this repair system include: ATPases hMSH2, hMSH6, hMSH3, hMLH1, hPMS2, hPMS1, and hMLH3 [27]. These genes code for proteins whose role is to detect and repair DNA replication errors that occur during mitosis. Mutation or methylation of the promoter region of MMR genes induces a deficiency in this repair system and mutations will accumulate, preferably at the level of microsatellites, regions of the genome particularly prone to replication errors. The cell acquires a “hypermutator” phenotype predisposing to the occurrence of mutations in certain oncogenes (pro-apoptotic BAX genes) or tumor suppressor genes (genes encoding the type II receptor of TGF β). The chronology of RER+ cancer mutations is poorly understood: the mutation of the type II TGF β receptor appears to be the earliest. In 2010, Earle et al. demonstrated an association between miRNA expression in CRCs with MSI subgroups, underlining post-transcriptional gene regulation by miRNAs [28].

The more recent work of Slattery et al. in 2016 tried to determine if there was a specific molecular pattern linked to specific expressions of particular miRNAs [29]. A total of 1893 CRC samples from patients based in the United States were analyzed. They found that MSI tumors have the majority of significantly different miRNAs expressions. Indeed, few different expressions of miRNAs by *TP53*, *KRAS*, CpG island methylator phenotype, and *BRAF* molecular phenotype for either colon or rectal carcinoma were highlighted. On the contrary, 94 miRNAs were differentially expressed between MSI and MSS tumors for colon carcinomas and 41 miRNAs for rectal carcinomas.

In 2003, Michael et al. highlighted the first implication of miRNAs in CRCs. This study showed a downregulation of miR-143 and miR-145 in CRC specimens. Interestingly, the authors did not observe altered levels of miRNA precursors, indicating a post-transcriptional regulation of their expression in CRC cells. The inhibited expression of miR-143 and miR-145 correlates with enhanced cancer cell proliferation [30]. MiR-145 inhibits the expression of multiple targets essential for sustained CRC growth such as insulin receptor substrate 1 (IRS-1), the proto-oncogene c-Myc, the proto-oncogene Yamaguchi sarcoma viral oncogen homolog 1 (YES-1), and signal transducers and activators of transcription 1 (STAT1) [31,32,33]. MiR-143 expression was found to negatively correlate with CRC metastasis [34]. MiR-143 inhibits key targets essential for CRC growth such as the GTPase KRAS, the DNA (cytosine-5)-methyltransferase 3A (DNMT3A), the receptor tyrosine-protein kinase erbB-3, and the mitogen-activated protein kinase 7 (MAPK7) [31,32]. In addition, other miRNAs regulators of CRC growth have been described. For example, overexpression of miR-21 was reported in CRC tissue (the clinical value of miR-21 will be discussed below) [35]. Also, miRNAs from the cluster miR-17-92 were found to be overexpressed in a broad spectrum of human cancers including CRCs. Using an engineered mouse model overexpressing this miRNA cluster, Huabin et al. demonstrated that these miRNAs inhibit CRC progression by targeting key inducers of cancer angiogenesis such as the vascular endothelial growth factor A (VEGFA) [36]. Other miRNAs, such as members of the let-7 family of miRNAs, are downregulated in CRCs [37] (the clinical value of let-7 will be reviewed below).

In 2006, Cummins et al. initially characterized the CRC miRNome. In the study, authors identified 133 miRNAs specific to CRC tissue compared to non-tumor tissue. MiR-143 and miR-145 were identified as parts of this CRC-specific miRNA signature [38]. In 2012, The Cancer Genome Atlas Network performed a comprehensive molecular characterization of CRCs [39]. The study conducted genome-scale analyses, including an miRNA expression profile, using 276 CRC samples that were compared to healthy mucosa. The expression of multiple miRNAs was found to be altered in CRCs as well as to correlate with CRC aggressiveness.

MiRNAs are thought to contribute to the initiation of CRCs. MiRNA-135a and miRNA-135b [25] are overexpressed in CRCs and inhibit the expression of the tumor suppressor adenomatous polyposis coli (APC), a key component in a cytoplasmic complex that inhibits the Wnt/β-catenin signaling pathway [40]. This pathway is aberrantly activated in CRCs and contributes to cell transformation upon APC mutation or loss of function [40]. Therefore, a role of miR-135a and miR-135b in the early steps of CRC oncogenesis could be speculated.

The transcription factor p53 is the most studied transcription factor and tumor suppressor. Generally, p53 activation is triggered as a response to DNA damage. Activation of p53 blocks cell cycle progression, thus allowing mechanisms of DNA repair to take place [41]. Deregulation of p53 expression was reported in ~60% of CRC samples [41]. A miRNA-related p53-mediated mechanism was reported in CRCs. Chang et al. discovered that p53 directly activates the transcription of miRNA-34a. MiRNA-34a is a tumor suppressor that triggers apoptosis and mimics the effects of p53 by regulating cell cycle progression, DNA repair, and angiogenesis [42]. 

The transcription factors zinc finger E-box binding homeobox 1 (ZEB1) and ZEB2 promote the epithelial to mesenchymal transition (EMT), a key process in the onset of metastasis. A mechanism of mutual inhibition linking ZEB1/2 to miRNA-200 was reported. ZEB1/2 inhibit miRNA-200 expression and miRNA-200 inhibits ZEB1/2. Therefore, an inhibited expression of miR-200 induces an accumulation of ZEB1/2 and correlates with enhanced CRC aggressiveness [43,44,45].

We performed a literature search within Pubmed between 2011 and 2021 with the keywords “miRNAs and colorectal cancer pathways” or “miRNAs and colorectal cancer signaling”. After finding the most relevant works aimed at demonstrating the role of some miRNAs targeting specific “signaling pathways or proteins” involved in the carcinogenesis of CRCs, we organized each publication below in Table 1.

## 4. MicroRNAs as a Diagnosis Biomarker in CRCs

The last decade witnessed key achievements in understanding the origin and functions of miRNAs. These studies pointed out the potential interest of miRNAs in clinical practice for patients in healthy or diseased conditions. Thus, they are linked to biological processes such as cell differentiation, development of the nervous system, muscle development, the cell cycle, cell signaling, immune response, angiogenesis, apoptosis, and autophagy [54]. On the other hand, we can highlight them within different pathological processes. Consequently, various fields of implementation of miRNAs within medicine emerged notably in terms of CRC biomarkers. Indeed, different studies have suggested that they could be appealing biomarkers for cancer through vesicular trafficking communication [55], in cardiology to diagnose heart failure [56], or more recently in neurology for the diagnosis and prognosis of neurodegenerative pathologies [57].

Performing a literature search with the keywords “miRNA and colorectal cancer and diagnostic biomarker” between 2011 and 2021 within Pubmed encompassing only clinical trials and randomized control trials in English, we were able to highlight 23 articles. After screening them, five had to be considered for review focusing on miRNAs as a diagnosis biomarker in CRCs.

They are summarized in the table below (Table 2).

First, miRNAs can help detect CRCs at an early stage and have become part of the screening process. As blood search within stools is currently widely used for CRC screening (fecal immunotest called “FIT”) [63], Wu et al. evaluated miRNA levels within stool samples [58]. The authors showed that miRNA-92a stool levels for patients without cancers were lower than those with CCRs. Furthermore, miRNA-92a was also sensitive and specific for the detection between polyps (higher levels) and patients without CRCs. These results can be explained by the function of miRNA-92a, which is a member of the cluster miRNA-17-92 located at chromosome 13q13 [64]. More precisely, it generally promotes cell proliferation, leading to tumor progression, suppresses apoptosis of cancer cells, and is able to induce tumor angiogenesis. MiRNA-92a was found to be upregulated in CRCs. It interacts with its target PTEN (phosphatase and tensin homolog) via the PTEN/PI3K (phosphoinositide 3-kinase)/Akt (protein kinase B) signaling pathway, triggering a decrease of E-cadherin expression, a biomarker of epithelial-to-mesenchymal transition (EMT) [65].

Precancerous lesions such as adenomas that are diagnosed in colonoscopies can also be detected based on the expression of miRNAs in serum as demonstrated by Zheng et al. [62]. In the study, the authors highlighted miRNA-191-5p and U6 as the most stable pair of reference genes (controls) in colorectal adenocarcinoma, colorectal adenoma, and healthy controls for serum microRNA qPCR analysis. Thus, using these controls, they demonstrated that serum miRNA-92a-3p was significantly higher in colorectal adenocarcinoma patients than colorectal adenoma patients and healthy controls (*p* < 0.001). Moreover, the difference in miRNA-92a levels was significant between colorectal adenoma patients and healthy controls (*p* < 0.001).

Secondly, miRNAs are great candidates for the diagnosis of CRCs such as different studies highlighted it irrespective of the sample type (serum or plasma) [59,60,61]. Both sensitivity and specificity reached high rates between 70% and 95%. It is also worth noting that in the last few years miRNAs have been sought within exosome-enriched fractions within serum [66]. Indeed, these nanovesicles are secreted by either normal or cancerous cells leading to the transport of different molecules such as proteins or RNAs [67]. As RNAs in exososmal cargo are drastically different between healthy and cancer-stricken patients, they can be indicators of the presence of transformed cells. The study of Ogata-Kawata et al. has shown that miRNA-23a, miRNA-1246, and miR-21 present a diagnostic accuracy for CRCs. The area under the curve (AUC) in receiver operating characteristic (ROC) curves were around 0.953, 0.948, and 0.798, respectively [68].

A recent meta-analysis [69] encompassing 35 studies published from 2009 to 2019 and including 3258 CRC patients and 2683 healthy controls analyzed serum-derived miRNA panels. More precisely, it focused on multiple miRNA serum-derived panels (and not single-miRNA assays), demonstrating them as promising markers of early diagnosis of CRCs. However, it is important to stress that there is a high degree of heterogeneity between studies: most studies integrated subjects from the Asian continent, some combined carcinoembryonic antigen (CEA) measures to miRNAs (which may improve diagnostic efficiency of early CRC detection).

## 5. MicroRNAs as a Prognosis Biomarker in CRCs (CRC Risk)

We searched within Pubmed “miRNA and colorectal cancer and prognosis biomarker” for articles between 2011 and 2021. Only clinical trials and randomized control trials in English were integrated. We found 20 research articles, which were carefully screened, resulting in 12 remaining studies for review focusing on miRNAs as prognostic and predictive biomarkers in CRCs (Table 3).

Regarding the prognosis, while the study of Schou et al. demonstrated higher rates of miR-345 in whole blood linked to shorter overall survival (OS) [73], the work of Hansel et al. [75] reported further findings. They showed that high tumor expression of miRNA-126 was significantly related to a longer progression-free survival (PFS). MiRNAs have thus different actions and links within the molecular signaling pathways of the initiation and maintenance of carcinogenesis. Consequently, their modifications’ rates show indirectly their effects on these different pathways and especially in terms of survival: for instance, miR-21 targets the genes involved in the PI3K pathway, which is downstream from the EGFR pathway regulating tumor growth, angiogenesis, and metastasis [81]. Thus, miR-21 reflects the KRAS signaling, and high levels are found in KRAS wild-type patients predicting increased response to anti-EGFR therapy (cetuximab) [82]. Furthermore, as described by the meta-analysis of Peng et al. [83], higher expression of miRNA-21 in patients with CRCs correlated with significantly shorter disease-free survival.

Due to the ever-increasing interest in exosomes, miRNAs derived from exosome-enriched fractions are used to define the prognosis of CRCs [84]. Beyond that, they are also able to distinguish chemoresistance and predict recrudescence. For example, Yagi et al. in 2019 found that plasma exosomal microRNA-125b may have been a monitoring biomarker of resistance to mFOLFOX6-based chemotherapy in 55 patients with advanced and recurrent CRCs. 

Targeted therapy has dramatically improved patients’ outcomes. However, drug resistance mechanisms leading to tumor recurrences remain a major clinical challenge [85]. Consequently, different studies have demonstrated the role of miRNAs as predictor markers of response to antiepidermal growth factor receptor (EGFR) monoclonal antibodies (cetuximab and panitumumab) [71,73,77,79], antiangiogenic therapy [74], chemotherapies (irinotecan, 5-fluorouracile) [78], and immunotherapy [76]. The main chemotherapy drugs used to treat CRCs are 5-fluorouracile and oxaliplatin. A study by Liu et al. [78] demonstrated the value of miR-21 in predicting response to 5-FU and oxaliplatin treatments. This was subsequently demonstrated by in vitro studies with CRC cell lines HT-29 [86]. MiRNAs can lead to high specificity when used in the development of personalized treatments, as was shown in the study of Sclafani et al. [80], which underlined such use with neoadjuvant treatment in the particular entity of rectal cancers.

In metastatic CRCs, clinical studies revealed a correlation between miRNA expression profiles and therapeutic response to EGFR- and vascular endothelial growth factor (VEGF)-targeted therapies.

Clinical studies of miRNAs in anti-EGFR therapy-treated CRC patients assessed only the miRNA expression in tumor tissue. In RAS wild-type metastatic CRC patients receiving anti-EGFR monoclonal antibody cetuximab and fluoropyrimidine-based therapy, upregulation of miR-31-5p and miR-31-3p correlated with worse progression-free survival. In the same setting of drug combinations and RAS wild-type metastatic CRC patients, decreased expression of miR-592 and miR-181a also correlated with worse PFS [87,88,89]. Furthermore, elevated expression of miRNA-140-5p and decreased expression of miR-1224-5p and miR-181a correlated with worse overall survival (OS) in these patients [87,89]. In KRAS-mutated and BRAF wild-type metastatic CRC patients receiving a cetuximab- and irinotecan-based third-line therapy, increased expression of miR-let-7a correlated with improved OS and PFS. This correlation was enhanced in patients with conserved miR-let-7a-binding sequence in KRAS-coding mRNA, suggesting that patients with high tumor expression of miR-let-7a may benefit from an anti-EGFR therapy irrespective of KRAS mutational status [90].

An original study revealed that in metastatic CRC patients receiving an anti-VEGF monoclonal antibody bevacizumab and capecitabine plus oxaliplatin therapy, overexpression of miR-664-3p and downregulation of miR-455-5p correlated with improved OS and PFS [91]. Subsequently, other clinical studies showed that elevated cancer tissue and plasmatic levels of miR-126 prior to bevacizumab therapy associated with improved therapeutic response [74,92]. Other circulating miRNA signatures were associated with improved response to bevacizumab. For example, an enriched basal plasmatic expression of miR-92b-3p, miR-3156-5p, miR-10a-5p, and miR-125a-5p predicted improved PFS in bevacizumab-treated metastatic CRC patients [93]. Moreover, elevated basal expression of miR-20b-3p, miR29b-3p, and miR-155-5p correlated with improved OS and PFS. However, a further increase of miR-155-5p expression at one month of bevacizumab treatment correlated with worse prognosis, highlighting an original prognostic value of the dynamic expression of circulating miRNAs in metastatic CRCs [94].

## 6. MicroRNAs as Therapeutics in CRCs

MiRNAs regulate the progression of various human diseases such as cancer, immune disorders, cardiovascular diseases, Alzheimer’s disease, and rheumatoid arthritis [95]. Recent studies highlighted different therapeutic miRNAs in several diseases. For instance, the liver-expressed miRNA-122, using the locked nucleic acid (LNA)–modified antisense oligonucleotide miravirsen, has been used in a phase II clinical trial as a potential treatment for hepatitis C viral infection (HCV) [96]. In this trial, 36 patients with chronic HCV genotype 1 infection were enrolled and randomized to three cohorts: 9 miravirsen-treated and 3 placebo-treated subjects in each cohort. They demonstrated that a four-week miravirsen treatment provides long-lasting suppression of the virus, with a good tolerance for patients with chronic HCV infection. 

In cancer, miRNAs regulate several cellular processes such as cell proliferation, invasion, and metastasis. There are two potential therapeutic ways to restore the physiological miRNA expression in tumor cells: (1) by inhibiting miRNA activity in case of oncogenic miRNA overexpression; (2) by rescuing an miRNA activity in the situation of a downregulated tumor suppressor miRNA. 

For the inhibition of miRNAs, different tools can be used such as locked nucleic acids (LNA), antisense anti-miR oligonucleotides (AMO), miRNA sponges, and miRNA antagomirs [97].

- Locked nucleic acids (LNA), or “bridged nucleic acids (BNAs)”, are modified RNAs with a 2’sugar modification and act as anti-miRNAs. Due to their structure, they have high stability and affinity [98],
- Antisense anti-miRNA oligonucleotides (AMO) are oligonucleotides with a complementary sequence used to neutralize miRNAs and their (dys)functions [99],
- MiRNA sponges, or “sponge-miR-mask technology”, are used to prevent several miRNAs from binding. However, these masks have low specificity regarding gene blockage [100],
- MiRNA antagomirs, or “anti-miRs “or “blockmirs”, are oligonucleotides designed to block molecules from binding to a specific site on miRNAs [101].

Conversely, for the restoration of miRNA activity, viral vectors expressing miRNAs or synthetic miRNA mimics [102] may be employed. However, while in specific cancers, such as mesothelioma, treatments based on miRNAs have been established [103,104], no clinical studies demonstrating miRNAs as effective therapeutics have been highlighted within the international literature on CRCs.

In 2016, Christopher et al. underlined the whole process from the discovery of a potential miRNA signature of a disease to clinical trials validating miRNA therapeutic development [105]. As a consequence, four steps have to be followed:

- Identification of miRNA signatures of a specific disease.
- Validation of miRNA signatures within gain- and loss-of-function studies in vitro and in vivo.
- Pharmacologic analysis of in vivo delivery studies with pharmacodynamic and pharmacokinetic analysis.
- Efficacy and toxicity analysis at a large scale within human clinical trials (phase I to phase III) aimed at approval for therapeutic use.

In the published literature on CRCs, only in vitro and in vivo studies have been found. 

Akao et al. examined the miR-143 and miR-145 expression levels in 63 CRC and 65 colon and rectum adenoma specimens with paired non-cancerous tissues [106]. Their expressions were shown to be downregulated in the early phase of carcinogenesis. Thus, with the clinical therapeutic goal, they chemically modified these miRNAs to increase their activity and stability. Injections of modified synthetic miR-143 were performed within nude-mice xenografted tumors derived from DLD-1 human CRC cells, directly first in the tumors and then in the second experiment into veins. Tumor sizes and weights decreased after these injections, demonstrating the tumor-suppressive effect of chemically modified synthetic miR-143 in vivo. Zhang et al. [107] demonstrated that miR-34a was downregulated in SW480 CRC cell lines. Transfection of the cells with miR-34a mimic showed, consequently, attenuation of both cell migration and invasion.

Previous in vitro studies have the stressed pro-apoptotic and anti-proliferative roles of miR-145 [108] and repression activity of miR-33a on the proto-oncogene Pim-1 action thus as a tumor suppressor inhibiting cell-cycle progression [109]. Consequently, Ibrahim et al. [110], in 2011, delivered these two miRNAs through a mouse model to validate their therapeutic interest. They used a specific method of delivery with a polyethylenimine (PEI)-mediated delivery of unmodified miRNAs. It resulted in reduced tumor proliferation and increased apoptosis through these chemically unmodified miRNAs complexed with PEI. More recently, Karimi et al. [111] restored miR-143 expression [34] by transfection of the pCMV-miR-143 vector into the SW480 CRC cells. Viability and migratory potential decreased after such a transfection. Hejazi et al. [112] combined a treatment with miR-193a mimics (miR-193a-5p known to be downregulated in CRCs and correlated with advanced stages [113]) and Taxol (antimicrotubule agent) in HT-29 CRC cell lines. Inhibition of migration and colony formations was observed, suggesting miR-193a replacement combined with Taxol chemotherapy as a potential treatment for CRCs.

More recently, in vitro studies have tried to tackle the topic of chemoresistance. Thus, Liu et al. [114], transfected oxaliplatin-resistant CRCs and normal intestinal fetal human cells with an miR-128-3p expression lentivirus. They conducted both in vitro and in vivo studies, highlighting an improvement in oxaliplatin response. Sun et al. [115], in 2019, infected DLD-1 and Caco-2 CRC cells with lentiviruses carrying miR-302a or targeted miR-302a (anti-miR-302a). When miR-302a was overexpressed, both migration and invasion were decreased with restoration of cetuximab response contrasting with cells expressing an increase in miR-302a downregulation.

Therefore, miRNAs appear to be attractive targets to treat CRCs, whatever the stage of the disease and considering its molecular specificities such as resistance to conventional chemotherapies use. A lot of patents from Europe and the USA have been issued miRNA treatments in the past the last decade. However, as indicated above, these treatments have focused on viral infections such as HCV (US Patent No. 7,307,067) and glioblastoma (United States Patent 8778903) [116]. We hope that in the next few years, following the interesting in vitro studies that we described earlier, a multiplication of clinical trials will be initiated. It will be easier as administration modes of miRNAs with effective pharmacodynamic and pharmacokinetic properties have improved [117]. Systemic delivery can be performed, but also intratumor injections with increased specificity and efficacy, with a minimization of side effects [118] (Table 4).

Indeed, thanks to both bioavailability and decreased toxicity, the intratumoral injection of miRNAs can be more effective than systemic delivery. One main advantage of local delivery is minimal nonspecific uptake by healthy organs avoiding toxicity and immunogenicity. The polyethylenimine (PEI)-mediated local application of miR-145 demonstrated antitumor effects in CRC mouse models [110]. Similarly, intratumoral injection of nanoparticles of siRNAs demonstrated efficiency in CRC nude-mice xenografts [119]. Nevertheless, local administration can be difficult at a human level and needs exploration, especially at early disease stages with the possible use of endoscopy. Moreover, it is important to underline that this strategy seems at first glance limited to the localized and accessible tumor. Thus, when CRCs are spreading, systemic delivery has to be assessed. This route delivery should protect miRNAs from early degradation in the blood circulation. In addition, it has to carry them to the target cells and facilitate cellular uptake without inducing any immunogenic response [120]. Thus, oligonucleotide editing [121] and systemic miRNA delivery systems such as LNAs, which are chemically modified RNA nucleotide analogs with high nuclease resistance [122], have been examined and are still being explored.

## 7. Conclusions

In last decade, miRNAs have shown increasing value as potential diagnostic and prognostic biomarkers in CRCs. Different studies both at fundamental and clinical levels are the basis of these advances. It can be highlighted that there is no one but different couples of miRNAs allowing these functions. However, analyses at a large scale are necessary to show with both sensitivity and specificity which couple has to be used in daily clinical care.

More precisely, miRNAs are major post-transcriptional regulators. Their deregulation participates in the process of colorectal carcinogenesis. To date, the use of miRNAs as diagnostic biomarkers for the screening of CRCs appears promising but requires further research. The deregulation of some miRNAs can identify colorectal tumors at risk of recurrence. Moreover, some deregulated miRNAs take part in chemoresistance mechanisms. The identification of miRNA biomarkers of resistance to anti-EGFR or anti-VEGF antibodies would allow for the optimization of the management of patients with metastatic CRCs. The therapeutic use of miRNAs is a very recent field of application. Different clinical trials have highlighted their impact in treating lung cancer, glioblastoma, or breast cancer but not yet colorectal cancers. Moreover, to be completely efficient, several optimizations are required. 

Challenges are to be considered and taken into consideration toward specificity, stability, pharmacodynamics and pharmacokinetics, immune activation, and efficacity toxicity in both in vivo and in vitro studies. Another important challenge in the next few years will be to integrate miRNAs within the therapeutic arsenal of personalized-medicine strategies for treatment of CRCs.

## Figures and Tables

**Table 1:** MiRNAs and CRC pathways.

MiRNA(s)	Sample	Signaling Pathways Involved or Proteins Involved		Role in Oncogenesis	Reference
MiRNA-10b	Tissue	E cadherin protein		Activates metastasis, vascular invasion, and tumor differentiation	Abdelmaksoud-Dammak et al. [46]
MiRNA-638	Tissue	TSPAN1 protein		Inhibits cell proliferation, invasion, and arrests the cell cycle in G1 phase	Zhang et al., 2014 [47]
MiRNA-17, 19, 20 and 9	Tissue	TGFβ-signaling		Cell proliferation	Pellat et al., 2018 [29]
MiRNA-21-5p	Tissue	Hippo signaling, Wnt signaling RAS signaling PI3K-AKT signaling TGF-β signaling Mismatch repair signaling	Erb signaling p53 signaling	Cell proliferation and cancer progression	Falzone et al., 2018 [48]
MiRNA-195-5p			MAPK signaling		
MiRNA-497-5p			p53 signaling FoxO signaling mTOR signaling MAPK signaling		
MiRNA-217	Tissue	MAPK signaling		Cell growth Apoptosis	Zhang et al., 2016 [49]
MiRNA-103a MiRNA-1827 MiRNA-137	Tissue	Wnt signaling		Cell cycle Apoptosis	Fasihi et al., 2018 [50]
MiRNA-106a	Tissue	ATG7 protein		Autophagy	Hao et al., 2017 [51]
MiRNA-182 and miRNA-135b	Tissue	PI3K-AKT signaling		Migration, adhesion, invasion, proliferation, and tumor angiogenesis	Jia et al., 2017 [52]
MiRNA-181a	Tissue	E cadherin protein		Cell motility, invasion, tumor growth	Ji et al., 2014 [53]
MiRNA-125a-3p	Tissue	PI3K-AKT signaling		Proliferation, migration, invasion, and angiogenesis	Liang et al., 2017

**Table 2:** MiRNAs as diagnostic biomarkers.

MiRNA(s)	Sample	Aim	Limit of Detection (LOD) [Unit]	Sensibility	Specificity	Reference
MiRNA-92a(miR-21)	Stools	Early diagnosis (screening)Diagnosis	435 [copies/ng]	71.6% for CRC 56.1% for polyp	73.3% for CRC and polyp	Wu et al., 2012 [58]
Six- miRNA signature: miRNA-21, let-7g, miRNA-31, miRNA-92a, miRNA-181b, miRNA-203	Serum	Diagnosis	9.595 [“of risk score function”]	96.4%	88.1%	Wang et al., 2014 [59]
MiRNA-21	Serum	Diagnosis	1.49 [fold change: ratio of the changes between miRNA value of CCR patients and miRNA value of healthy patients]	77%	78%	Basati et al., 2014 [60]
MiRNA-21	Plasma	Diagnosis	0.00220 [expression relative, log 10 (2^−^^Δ^^Ct^)]	76.2%	93.2%	Du et al., 2014 [61]
MiRNA-191-5p	Serum	Reference gene for diagnosis	Not reported	Not reported	Not reported	Zheng et al., 2013 [62]

**Table 3:** MiRNAs as prognostic biomarkers.

MiRNA(s)	Sample	Aim	Results	Reference
MiRNA-125b-1 MiRNA-378a	Tissue	To predict efficacy of vaccine (5-peptide combination) against CRCs	+ After peptide vaccines combined with oxaliplatin-containing chemotherapy were given: miR-125b-1 in cancer cells (*p* = 0.040), and miR-378a in both cancer cells (*p* = 0.009) and stromal cells (*p* < 0.001) were negatively associated with OS	Tanaka et al., 2017 [70]
MiRNA-31-3p	Tissue	Predictive biomarker of selection for anti-EGFR mAbs.	+ Low miR-31-3p expression linked to better overall response rate	Anandappa et al., 2019 [71]
MiRNA-100	Tissue	Prognostic	+ Its downregulation showed poor overall survival (OS)	Chen et al., 2014 [72]
MiRNA-345	Whole blood	Prognostic Predictive about cetuximab and irinotecan response	+ −MiR-345, single prognostic biomarker for both OS and progression-free survival (PFS) −High miR-345 expression was associated with lack of response to treatment with cetuximab and irinotecan	Schou et al., 2014 [73]
Circulating microRNA-126 (cir-miRNA-126)	Plasma	Predictive of anti-angiogenic treatment resistance	+ Non-response to anti-angiogenic treatment was linked to increase of cir-miRNA-126	Hansen et al., 2015 [74]
MiRNA-126	Tissue	Prognostic	+ High tumor expression of miRNA-126 was significantly related to a longer PFS	Hansen et al., 2013 [75]
MiRNA-6826 and miRNA-6875	Plasma	Predictive to vaccine treatment response	+ Plasma miR-6826 and miR-6875 may be predictive biomarkers for a poor response to vaccine treatment	Kijima et al., 2017 [76]
MiRNA-31-5p	Tissue	Predictive response to cetuximab and panitumumab in metastatic wild-type KRAS colorectal cancer patients in progression after cetuximab in combination with irinotecan-based chemotherapy (FOLFIRI or irinotecan alone) who received panitumumab monotherapy	+/− −Predictive for cetuximab response −Non-predictive for panitumumab response	Kiss et al., 2016 [77]
MiRNA-21	Tissue	Predictive response to neoadjuvant chemotherapy (FOLFOX4) for locally advanced CRC (staging cT3–4, any N, M0 or cT2, N1)	+ Cut-off: 10.32 for differentiating pathological responders from non-responders, with a sensitivity of 80.0% and specificity of 88.2%	Liu et al., 2011 [78]
MiRNA-31-3p	Tissue	Predictive of cetuximab therapy efficacy for patients with *RAS* WT mCRC.	+ Low miR-31-3p expressers significantly benefited from cetuximab compared with bevacizumab for PFS, OS in multivariate analyses	Laurent-Puig et al., 2019 [79]
Single nucleotide polymorphism (rs61764370, T > G base substitution) in the let-7 complementary site 6 (LCS-6) of KRAS miRNA	Tissue	Rs61764370 predictive of neoadjuvant chemoradiotherapy for locally advanced rectal cancer	+ Randomized phase II trial of neoadjuvant CAPOX (capecitabine + oxaliplatin) followed by chemoradiotherapy, surgery, and adjuvant CAPOX plus or minus cetuximab in locally advanced rectal cancer:carriers of the G allele had a statistically significantly higher rate of complete response (CR) after neoadjuvant therapy and a trend for better 5-year PFS and OS rates. Both CR and survival outcomes were independent of cetuximab use. The negative prognostic effect associated with KRAS mutation appeared to be stronger in patients with the LCS-6 TT genotype compared to those with the LCS-6 TG genotype	Sclafani et al., 2015 [80]

+: aim of the study achieved; +/−: aim of the study partially achieved; −: aim of the study not achieved.

**Table 4:** Potential approaches for miRNA therapeutic delivery.

Delivery System	MiRNA(s)	MiRNA Type	Mode of Delivery	CRC Subtype	Target Gene	Reference
Polyethylenimine	MiR-145 MiR-33a	Double-stranded RNA (dsRNA)	Intraperitoneal Intravenous	CRC	c-Myc, ERK5	Ibrahim et al., 2011 [110]
Carbonate apatite	MiRNA-4689	Mature-miR	Intravenous	KRAS-mutant CRC	KRAS, AKT1	Hiraki et al., 2015 [123]
Carbonate apatite	MiRNA-29b-1-5p	Mimic-miR	Intravenous	KRAS-mutant CRC	BCL-2, MCL1	Inoue et al., 2018 [124]
Exosome	MiRNA-143	Chemically modified RNA molecules (BP-miR) entrapped by MVs (microvesicules)	Intravenous	CRC	None	Akao et al., 2011 [125]
Atelocollagen	MiRNA-34a	Precursor-miRNA (pre-miRNA or pre-miR)	Subcutaneous	CRC	E2F	Tazawa et al., 2007 [126]
